# Fatal interstitial lung disease associated with rituximab in diffuse large B-cell lymphoma: A case report and literature review

**DOI:** 10.1097/MD.0000000000046554

**Published:** 2025-12-12

**Authors:** Li Li, Fei Liu, Jie Zhou, Xiang-Lei Chen

**Affiliations:** a Department of Respirology, Yidu Central Hospital of Weifang, Qingzhou, Shandong Province, China; b Department of Hematology, Yidu Central Hospital of Weifang, Qingzhou, Shandong Province, China.

**Keywords:** fatal, interstitial lung disease, rituximab, TNF-α

## Abstract

**Rationale:**

Rituximab-associated interstitial lung disease (ILD) may initially be asymptomatic, and serial monitoring of serum tumor necrosis factor-alpha levels may facilitate timely diagnostic intervention with pulmonary computed tomography.

**Patient concerns:**

A 73-year-old patient with diffuse large B-cell lymphoma (DLBCL) developed ILD during treatment with a rituximab-containing regimen.

**Diagnoses:**

Rituximab-associated interstitial disease was made.

**Interventions:**

The patient was treated with corticosteroids, intravenous immunoglobulin, and antifibrotic therapy.

**Outcomes:**

The ILD progressed, and the patient ultimately succumbed to type I respiratory failure.

**Lessons:**

Clinicians should remain vigilant for rituximab-associated ILD, which, although uncommon, can progress rapidly and result in poor outcomes.

## 1. Introduction

Rituximab is a monoclonal antibody targeting CD20, primarily used in the treatment of CD20-positive B-cell lymphomas in hematologic disorders. Infusion-related reactions and hepatitis B virus reactivation are well-known adverse effects recognized by clinicians. Interstitial lung disease (ILD) was reported as a relatively rare adverse event during clinical trials of rituximab. However, post-marketing experience has indicated that rituximab-associated ILD occurs more frequently than reported in clinical trials and may be potentially fatal.^[1]^

## 2. Case presentation

**Figure 1.**
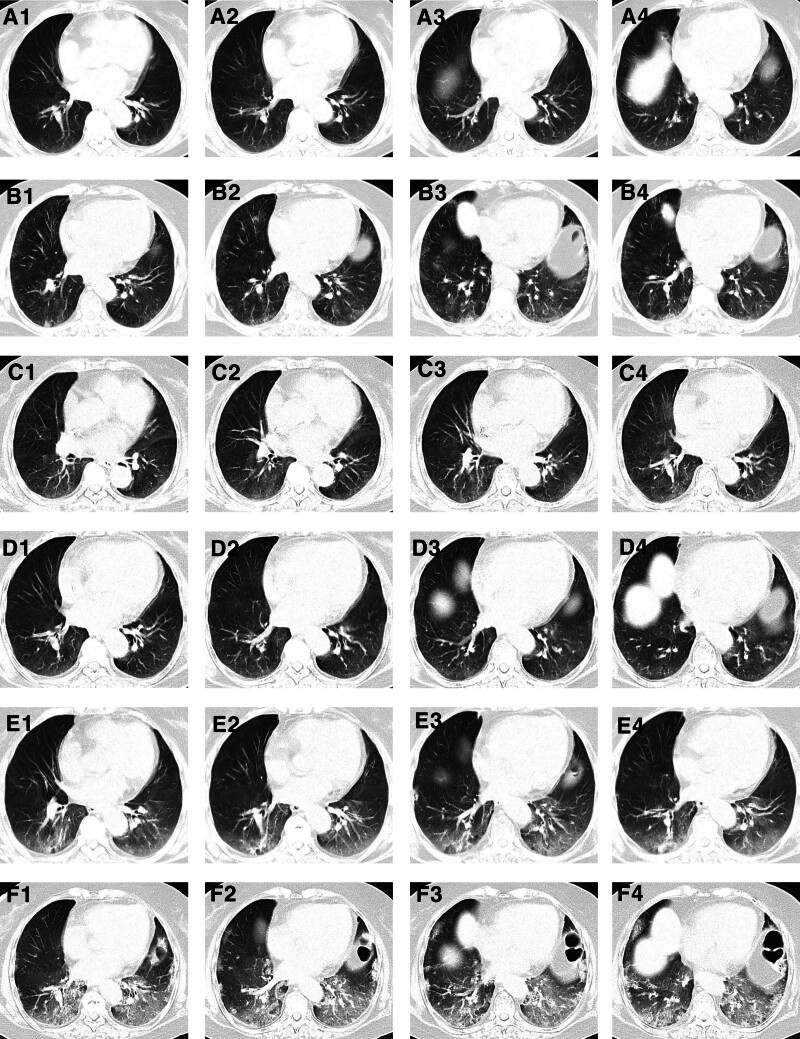
Rituximab-associated lung injury. (A) Before rituximab therapy. (B) After 4 cycles of rituximab treatment. (C) Five days after methylprednisolone therapy. (D) Fourteen days after methylprednisolone treatment. (E) When methylprednisolone was tapered to 12 mg. (F) Progressive worsening of pulmonary injury.

**Figure 2.**
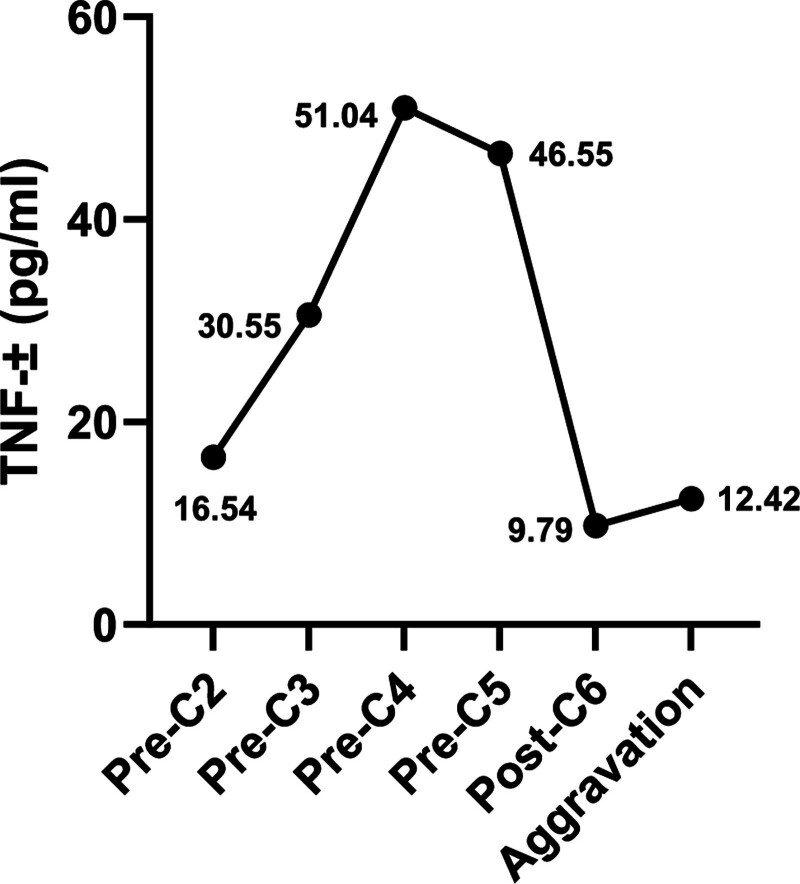
Changes in TNF-α concentration during rituximab therapy (reference range: 0–8.5 pg/mL). TNF-α = tumor necrosis factor-alpha.

A 73-year-old female presented with a sensation of a foreign body in the pharynx. She underwent right tonsillectomy under endoscopy. Postoperative pathology, combined with imaging studies including computed tomography (CT) scans of the neck, chest, abdomen, and pelvis, along with peripheral blood smear, bone marrow aspiration, and biopsy, confirmed the diagnosis of diffuse large B-cell lymphoma (DLBCL), stage IIA with an international prognostic index score of 1 (low risk), involving the Waldeyer ring and cervical lymph nodes. The patient had a past medical history of proteinuria, and renal ultrasonography revealed bilateral renal parenchymal damage. However, the estimated creatinine clearance rate remained above 70 mL/min. The patient was started on R-CHOP (rituximab 600 mg on day 0; vincristine 2 mg, cyclophosphamide 1200 mg, pirarubicin 50 mg on day 1; prednisone 100 mg on days 1–5) immunochemotherapy. After the first 2 cycles, she developed neutropenia. Therefore, R-COP (rituximab, cyclophosphamide, vincristine, and prednisone; excluding pirarubicin) was administered for cycles 3 and 4. Chest CT prior to the first cycle containing rituximab showed no lung abnormalities (Fig. 1A). Before the fifth cycle, chest CT revealed multiple patchy and flocculent high-density opacities in both lungs, predominantly located in the subpleural regions of the posterior lower lobes. Some lesions showed fine reticular changes with ill-defined margins (Fig. 1B). Based on the temporal relationship and radiologic features, the changes were suspected to be rituximab-associated ILD. R-COP was thus discontinued, and subsequent cycles 5 and 6 were conducted with COP (cyclophosphamide, vincristine, and prednisone) chemotherapy only. Serum tumor necrosis factor-alpha (TNF-α) levels were monitored before each cycle (Fig. 2). Although the patient had no respiratory symptoms prior to the fifth cycle, progressive elevation in TNF-α prompted a chest CT, which identified ILD. Suspecting rituximab-associated ILD, the patient was started on methylprednisolone 80 mg daily, followed by gradual tapering. During the 5th and 6th cycles of COP chemotherapy, the patient remained asymptomatic. Follow-up chest CT showed gradual resolution of the interstitial lesions (Fig. 1C and D), and TNF-α levels normalized (Fig. 2). However, when the methylprednisolone dose was tapered to 12 mg daily, the patient developed cough, chest tightness, and dyspnea. Repeat chest CT demonstrated progression of ILD, although TNF-α levels had not increased significantly (Fig. 2). Infectious etiologies were carefully excluded through repeated sputum cultures and microbiological testing, all of which were negative for bacterial, viral, or fungal pathogens, including opportunistic organisms. Methylprednisolone was re-escalated to 80 mg daily but showed limited efficacy. Intravenous immunoglobulin (IVIG) and nintedanib were added, but the ILD continued to progress (Fig. 1E and F), ultimately leading to type I respiratory failure requiring ventilatory support. Oxygenation parameters indicated hypoxemia consistent with type I respiratory failure in the terminal stage. The patient eventually died of respiratory failure. A timeline table (Table 1) summarized the treatment cycles, TNF-α levels, and corresponding CT findings.

**Table 1:** Timeline table summarizing treatment cycles, TNF-α levels, and CT findings.

Cycle	Time	Regimen	Cumulative R dosage	TNF-α Monitoring	TNF-α (pg/mL)	CT monitoring	CT findings
C1	2024/9/22	R-CHOP	600 mg	Before-C2	16.54	Before-C1	No abnormalities (Fig. 1A)
C2	2024/10/15	R-CHOP	1200 mg	Before-C3	30.55	Before-C5	Interstitial changes (Fig. 1B)
C3	2024/11/5	R-COP	1800 mg	Before-C4	51.04	After-C5	Partial resolution (Fig. 1C)
C4	2024/11/27	R-COP	2400 mg	Before-C5	46.55	Before-C6	Resolution continues (Fig. 1D)
C5	2024/12/18	COP	2400 mg	After-C6	9.79	1 mo after C6	ILD progression (Fig. 1E and F)
C6	2025/1/8	COP	2400 mg	Aggravation	12.42	2.5 mo after C6	ILD progression (Fig. 1E and F)

C = cyclophosphamide, CT = computed tomography, H = hydroxydaunorubicin, O = Oncovin (vincristine), P = prednisolone, R = rituximab, TNF-α = tumor necrosis factor-alpha.

## 3. Treatment

The therapeutic approach in this case was guided by expert consensus.^[2,3]^ Initial treatment included methylprednisolone 80 mg daily, tapered to 40 mg after one week, followed by weekly dose reductions of 4 mg. Upon reaching a dose of 12 mg daily, the patient developed progressive chest tightness and dyspnea. Repeat chest CT revealed worsening ILD. Methylprednisolone was increased back to 80 mg daily, and treatment was intensified with IVIG 0.4 g/kg/day for 5 consecutive days and nintedanib 150 mg twice daily.

## 4. Discussion

Pulmonary toxicity induced by antineoplastic agents may be cumulative dose-dependent; however, it is more often agent-specific and unpredictable in occurrence. A study based on the FDA Adverse Event Reporting System database indicated that rituximab is among the drugs most commonly associated with ILD.^[4]^ The mechanism of rituximab-induced ILD remains unclear, but may be related to complement-dependent cytotoxicity, antibody-dependent cellular cytotoxicity, and increased expression of cytokines such as TNF-α,^[5,6]^ interleukin-6 (IL-6),^[7]^ and interleukin-1β (IL-1β).^[8]^ In this case, serum TNF-α levels were routinely monitored prior to rituximab administration. A progressive increase in TNF-α levels was observed, prompting chest CT evaluation even in the absence of respiratory symptoms. ILD was detected, leading to discontinuation of rituximab and initiation of methylprednisolone therapy with gradual tapering. As radiological findings of ILD improved, serum TNF-α levels concurrently declined to within the normal range, suggesting a possible role of TNF-α in the pathogenesis of rituximab-induced ILD. However, this observation is exploratory and preliminary, derived from a single case, and should be interpreted with caution until confirmed in larger cohorts.

Liote et al conducted a systematic review and literature analysis and classified rituximab-induced pulmonary injury into 3 types^[9]^: Early-onset hyperacute pulmonary injury, which may occur within hours after the initiation of rituximab infusion. It often presents as acute respiratory distress syndrome and is thought to be associated with high tumor burden and tumor lysis during therapy; Acute or subacute pulmonary injury, which typically arises after the 3rd or 4th treatment cycle. Radiologically, it may present as ground-glass opacities or diffuse/localized alveolar infiltrates. This pattern is suspected to result from a hypersensitivity reaction to rituximab; and Delayed-onset pulmonary injury, which generally occurs more than 8 weeks after the last rituximab infusion. It may be related to the prolonged retention of rituximab in the body and immune reconstitution. In addition to prolonged rituximab retention, other factors may contribute to delayed onset and variable severity of ILD. Differences in individual immune responses, particularly in cytokine release such as TNF-α, IL-6, and IL-1β, may influence the timing and intensity of lung injury.^[10]^

In the present case, ILD was detected following the fourth cycle of therapy. Initial treatment with methylprednisolone was effective, but ILD worsened during steroid tapering. At that time, however, serum TNF-α levels remained within the normal range, suggesting that multiple mechanisms may be involved in rituximab-induced ILD. Other potential causes of ILD cannot be completely excluded. Cyclophosphamide itself is not directly pneumotoxic; however, some of its metabolites may exhibit pneumotoxic effects, although a clear dose-response relationship has not been established. Pulmonary toxicity from cyclophosphamide is thought to result from genetically determined differences in local pulmonary metabolism of the drug.^[11]^ Several studies^[9,12]^ also support that lung injury occurring during rituximab-containing chemotherapy for lymphoma is most likely attributable to rituximab. Therefore, in this patient, cyclophosphamide-induced ILD is considered unlikely. Studies have suggested that the use of granulocyte colony-stimulating factor (G-CSF) as supportive therapy during R-CHOP–like regimens may increase the risk of ILD. In the present case, the patient also received G-CSF for chemotherapy-induced neutropenia, which may have contributed to the development of ILD.^[13]^ Carcinomatous lymphangitis refers to tumor infiltration of the lymphatic vessels, most commonly involving the lungs. It usually occurs in the terminal stages of malignancy. Radiographic and CT findings typically include reticular, nodular, or reticulonodular opacities with thickening of the bronchovascular bundles. However, carcinomatous lymphangitis is most often caused by primary tumors of the breast, lung, stomach, colon, or prostate. ILD secondary to carcinomatous lymphangitis from diffuse large B-cell lymphoma (DLBCL) is rare. Bronchoalveolar lavage (BAL) is generally not diagnostic for chemotherapy-induced pulmonary toxicity, but it plays an important role in ruling out alternative causes such as infections or pulmonary tumor infiltration, thereby narrowing the differential diagnosis.^[14]^ BAL was not feasible in our patient due to her clinical condition. Similarly, surgical lung biopsy, while considered the gold standard for confirming ILD, carries significant procedural risks in frail patients and is rarely performed in clinical practice. In this case, although BAL was not performed, repeated sputum cultures since the onset and progression of respiratory symptoms yielded no evidence of bacterial, viral or fungal infection, including opportunistic pathogens. Rituximab-induced ILD is commonly associated with restrictive ventilatory dysfunction and reduced diffusing capacity for carbon monoxide (DLCO) on pulmonary function testing.^[15]^ However, in this patient, pulmonary function testing could not be performed due to progressive clinical deterioration. This represents an important limitation, as the absence of PFTs restricted our ability to objectively quantify the severity of respiratory dysfunction. There are also case reports describing patients with rituximab-induced ILD who initially responded well to corticosteroids, but later experienced deterioration during steroid tapering, with no response to subsequent steroid escalation, and even death.^[16]^ Therefore, in this patient, the progression of ILD is still considered to be associated with rituximab. The largest study to date on rituximab-associated interstitial lung disease (R-ILD) has demonstrated that high-resolution CT, bronchoalveolar lavage combined with metagenomic next-generation sequencing (mNGS), and lung biopsy are the main diagnostic and differential tools. R-ILD exhibits substantial heterogeneity in its clinical presentation. Corticosteroids remain the mainstay of treatment, with tapering recommended at a rate of 2% per week if high-resolution CT findings show improvement.^[17]^ In some cases, rituximab rechallenge has been attempted after ILD resolution.^[17,18]^ In our patient, however, ILD initially improved only transiently before rapidly deteriorating, precluding any possibility of rituximab rechallenge. These divergent clinical outcomes highlight the considerable heterogeneity of R-ILD.^[17,18]^ In summary, the patient’s ILD is most consistent with rituximab-associated ILD. Other potential causes – including cyclophosphamide toxicity, infection, and carcinomatous lymphangitis – were considered and deemed unlikely based on clinical, microbiological, and imaging findings. Although BAL and pulmonary function testing were not feasible, the temporal relationship with rituximab, imaging evolution, and initial steroid response support a causal association.

The European Respiratory Society and the American Thoracic Society have proposed the concept of acute exacerbation of idiopathic pulmonary fibrosis (AE-IPF), and have acknowledged that patients with connective tissue disease-associated interstitial lung disease (CTD-ILD) may also experience acute exacerbations.^[19,20]^ The diagnostic and therapeutic course in this case suggests that rituximab-associated ILD may similarly be subject to acute exacerbation. Such exacerbations significantly increase patient mortality. Extensive research has been conducted on molecular biomarkers and therapeutic strategies for AE-IPF, with evidence suggesting that treatment with intravenous immunoglobulin (IVIG)^[21]^ and nintedanib^[22]^ during acute exacerbation may improve patient outcomes. However, in this case, combined therapy with IVIG and nintedanib during the acute exacerbation failed to improve the patient’s prognosis. Additionally, anti-TNF therapy has also been attempted in the management of rituximab-associated ILD, but clinical outcomes have generally been unsatisfactory.^[7]^

Interestingly, rituximab has also been employed as a therapeutic option in connective tissue disease–associated ILD and other refractory autoimmune lung disorders, where it may exert beneficial immunomodulatory effects. Paradoxically, rituximab itself has been implicated as a cause of ILD in lymphoma patients. We believe this contrast underscores the need for further mechanistic studies to delineate the context-dependent effects of rituximab on the lung.

Additionally, the fact that only a subset of patients develop rituximab-associated ILD suggests that host genetic background may also play a role in modulating susceptibility. Variants such as the MUC5B promoter polymorphism (rs35705950),^[23]^ telomerase-related genes (e.g., TERT),^[24]^ and loci including Toll-interacting protein (TOLLIP) gene^[25]^ have been linked to fibrotic ILD. These variants affect epithelial repair, telomere maintenance, and fibrotic signaling, and could plausibly increase vulnerability to drug-induced lung injury. Additionally, these genetic susceptibilities could explain why only a subset of patients develop severe or progressive disease. Although specific genetic associations with rituximab-induced ILD have not yet been demonstrated, current evidence supports evaluating these candidate variants in future studies to improve risk assessment and guide individualized prevention.

This study has several limitations. First, due to the patient’s compromised clinical condition, bronchoalveolar lavage could not be performed, precluding microbiological testing, cytological analysis, and cytokine profiling, and lung biopsy was not feasible to exclude carcinomatous lymphangitis. Second, only TNF-α was monitored, while a broader cytokine panel, including IL-6 and others, was not assessed, limiting our ability to comprehensively characterize cytokine abnormalities associated with rituximab-induced ILD. Finally, genetic analyses to evaluate potential susceptibility variants linked to R-ILD were not conducted.

## 5. Conclusion

Continuous monitoring of serum TNF-α levels prior to rituximab administration may help identify patients at risk of ILD; however, this observation is preliminary, and larger studies are required before clinical implementation can be recommended. Additionally, monitoring TNF-α alone is insufficient – other cytokines such as IL-6,^[7]^ and immune cell populations, including lymphocyte subsets, may also play a role in the pathogenesis of rituximab-associated ILD and warrant further investigation. Glucocorticoid therapy has demonstrated efficacy in treating rituximab-associated ILD, but disease relapse or exacerbation during the tapering process remains a concern. Therefore, it is essential to determine the optimal tapering strategy and to identify predictive biomarkers that can guide safer and more individualized glucocorticoid tapering.

Currently, there is no specific diagnostic method for rituximab-associated ILD. Imaging findings are nonspecific and may be confounded by differential diagnoses such as infection or carcinomatous lymphangitis. Hence, multicenter collaboration is needed to accumulate more clinical cases and to develop noninvasive or minimally invasive diagnostic approaches with higher specificity.

## Acknowledgments

This manuscript was translated with the assistance of ChatGPT (OpenAI), and the final version was thoroughly reviewed by the authors.

## Author contributions

**Conceptualization:** Li Li, Xiang-Lei Chen.

**Data curation:** Li Li, Xiang-Lei Chen.

**Formal analysis:** Fei Liu, Jie Zhou.

**Writing – original draft:** Li Li, Xiang-Lei Chen.

**Writing – review & editing:** Xiang-Lei Chen.
